# COVID-19 Vaccination Attitude and Behavior among Nurses at a West Texas Regional Hospital

**DOI:** 10.3390/vaccines11020343

**Published:** 2023-02-03

**Authors:** Christopher J. Peterson, Mostafa Abohelwa, Afrina Rimu, Drew Payne, Shengping Yang, Tammy Williams, Erin Nash Rowin, Kenneth Nugent

**Affiliations:** 1 Department of Internal Medicine, Virginia Tech Carilion School of Medicine, Roanoke, VA 24106, USA; 2 Department of Internal Medicine, Texas Tech University Health Sciences Center, Lubbock, TX 79430, USA; 3 Pennington Biomedical Research Center, Baton Rouge, LA 70808, USA; 4 Nursing Administration, University Medical Center, Lubbock, TX 79430, USA; 5 School of Nursing, Texas Tech University Health Sciences Center, Lubbock, TX 79430, USA

**Keywords:** vaccination, COVID-19, vaccine hesitancy, nurses

## Abstract

Vaccinations against COVID-19 infection have become a contentious issue in the United States. Multiple segments of society, including healthcare workers, have expressed concerns regarding the need for vaccination and the safety of current vaccines. Many hospital-based nurses have helped care for patients with severe COVID-19 infections. An anonymous online survey was sent to the nursing staff at University Medical Center in Lubbock, TX, USA, through a hospital-based email system to determine vaccination status and attitudes towards the COVID-19 vaccine and other routine vaccines. Multivariable regression analysis was used to determine factors associated with vaccination. A total of 251 nurses responded to this survey; 211 nurses (83.7%) had received the vaccine. Almost all nurses (242, 96%) had received all childhood vaccinations, and 231 (91.7%) had received an influenza vaccination in the prior year. A minority of nurses (75, 29.8%) supported mandatory vaccination for healthcare workers. The reasons for declining vaccination included the possibility that diet and alternative medications provided better protection against COVID-19. This survey demonstrates that over 80% of nurses working in a hospital managing very sick patients with COVID-19 infection had been vaccinated. However, nurses who did not take the annual influenza vaccine and did not consider other protective measures useful (such as mask-wearing) were significantly less likely to vaccinate. Nurses can provide an important resource for conversations with the public and patients about vaccine initiatives.

## 1. Introduction

The COVID-19 pandemic has emphasized the importance of vaccination and highlighted attitudes of vaccine hesitancy and skepticism. When the first SARS-CoV-2 vaccines became available in the United States in late 2020 and early 2021, healthcare workers (HCWs) were prioritized to receive the vaccine due to their greater exposure to COVID-19 and to prevent transmission of this illness to their patients and other healthcare workers [1,2]. Despite the severity of the pandemic, many healthcare workers were hesitant to vaccinate, with reports in early 2020 showing concerningly low vaccination rates [3]. While many hesitant HCWs expressed concern about vaccine safety and efficacy [4], it was hoped that more HCWs would choose to vaccinate over time when more data and personal experience with vaccinated colleagues might improve acceptance.

Aw et al. undertook a scoping review of vaccine hesitancy in high-income countries based on the literature available through March 2021 [5]. They identified four themes associated with vaccine hesitancy; these included vaccine-specific concerns, individual concerns, group concerns, and contextual-related factors. In this review, younger age, female gender, non-White ethnicity, and lower education were common contextual factors associated with increased hesitancy. The lack of recent influenza vaccination, a lower self-perceived risk of contracting COVID-19, lesser fear of COVID-19, believing that COVID-19 is not severe, and not having chronic medical conditions were frequently studied group and individual factors associated with hesitancy. Vaccine-specific factors included the belief that the vaccines are not safe or effective and increased concern about the rapid development of COVID vaccines. The authors concluded that these factors or determinants need to be considered when developing policies regarding vaccination.

These authors subsequently reported results from an online, anonymous survey conducted between March and July 2021 of all staff in three community hospitals in Singapore [6]. Based on logistic regression analysis, female gender, younger age, not having a loved one or friend infected with COVID-19, and obtaining information from newspapers were associated with vaccine hesitancy. Vaccine hesitancy was lower in physicians and nurses than in hospital administrative staff and allied health staff. The authors noted that vaccine hesitancy is a complex decision based on the interplay of multiple factors and that efforts to improve vaccine uptake would require several approaches. Both these studies collected information in the early phase of vaccine development, distribution, and administration and follow-up studies to identify important considerations when studying vaccine acceptance and uptake in healthcare workers.

Vaccine mandates, whether government or employer-based, were also used to increase acceptance. However, as of this writing and more than two years into the pandemic, some HCWs continue to remain unvaccinated against COVID-19 [7]. While rates of HCW vaccination have improved with time [3], there are still some HCWs who are resistant. This may be problematic as it can place HCWs at a greater risk of illness [8], could produce greater strain on healthcare resources due to absenteeism [9], and fails to limit the spread of SARS-CoV-2 to others [10]. Healthcare workers are one of the most trusted patient resources regarding vaccination [11], and vaccine hesitancy from HCWs may exacerbate public vaccine hesitancy. Indeed, it can seem puzzling that individuals who have training in medical sciences, work at institutions that have historically required vaccination as part of employment, and care for ill patients would refuse a vaccine that has been demonstrated to have high safety and efficacy. However, historical attitudes about vaccination, even among HCWs, are varied and complex, and attitudes regarding COVID-19 vaccination have proven no different [12]. If vaccination rates among HCWs are to be improved, their reasons for vaccine hesitancy, including their attitudes towards non-COVID-19 vaccines, should first be assessed.

This survey involves the nursing staff at a West Texas regional referral hospital to better understand the attitudes and behaviors regarding COVID-19 vaccination. This hospital employs approximately 1650 nurses based on nursing manager reports. During the pandemic, 901 nurses tested positive for COVID-19 infection. This resulted in 4505 lost workdays (54,060 h). Some nurses also missed work after being in quarantine because of recent close contact with infected persons. The hospital provided multiple vaccine fairs and education sessions for the nurses and hospital staff to provide education and promote vaccination; it provided onsite vaccination. Over 80% of the nurses were eventually vaccinated. However, multiple nurses and other hospital employees applied for vaccine exemption; most of these requests were based on religious reasons. All cases were reviewed by a small panel that included a chaplain, a physician, and a legal representative. Eventually, 1298 exemptions were granted, and two workers resigned. Approximately 1154 patients were hospitalized in the medical intensive care unit in this hospital between March 2020 and March 2022; multiple other patients were hospitalized on inpatient non-ICU medical services (information provided by M Funderburk, CEO, UMC Health System, Lubbock, TX, USA, 16 December 2022).

## 2. Materials and Methods

We conducted an anonymous online survey of nursing staff that was sent and available to nurses between 28 February 2022 and 28 March 2022. This survey was approved by the Texas Tech University Health Sciences Center Institutional Review Board (L22-075). The initial draft for this survey was based on a publication based on a multicenter health worker survey in Canada [10] and on the authors’ experience with surveys distributed to medical students, residents, faculty, and staff at this Health Sciences Center [11,12,13]. Two authors (TW and ENR) then reviewed the survey to make certain that all questions were defined objectively and applicable to nursing participants. They expanded the distribution list to include nurses with other credentials, such as a nursing diploma and LVN certification. The survey was then distributed to five nurses who reviewed the survey and provided feedback regarding questions and clarity. The final, revised survey (Appendix A) was then distributed through a hospital email system; a reminder email was sent 2 weeks after the initial distribution. The distributed information was confidential, and participants were permitted to terminate their participation at any time. Qualtrics (https://www.qualtrics.com/; accessed 1 December 2021) and Excel were used to collect and analyze survey data. The survey pool size was estimated to be 1650 nurses based on nursing manager reports. The survey sample size calculation was based on a total population of 1650 and a projected 20% non-vaccinated rate; the estimate was 215 respondents with 95% confidence and a 5% margin of error. All the nurses employed at the hospital received the survey. If everyone who received the survey completed it, then with a projected 20% non-vaccination rate at a 95% confidence level, the margin of error for estimating the non-vaccination rate would be 2%. However, with a low response rate, the margin of error is 5%.

Incomplete responses, where the participant did not complete the survey, were removed. Descriptive statistics were used to describe the characteristics of the study participants. Categorical variables were summarized as frequencies, and continuous variables were summarized using means and standard deviations or medians and ranges, as appropriate. Simple logistic regression was used to evaluate the association between a risk factor and receipt of COVID-19 vaccine. Multiple-variable logistic regression was used to evaluate such an association while adjusting for all other risk factors. The statistical significance level was set at 0.05. Multiple testing adjustment was not performed. All analyses were performed using SAS (Windows version 9.3; SAS Institute, Cary, NC, USA) and the statistical program R version 4.1.3 (https://cran.r-project.org/; accessed 1 October 2022). Minor changes to free text responses were made to ensure anonymity.

## 3. Results

A total of 251 nurses responded to the survey (Table 1). The majority of respondents (211/251; 83.7%) had received the vaccine. Attitudes and behaviors regarding vaccination were generally supportive. The majority of respondents had received at least some childhood vaccinations (248; 98.4%), and most (242; 96%) received all of them. The majority also supported vaccination against other diseases (236/251; 93.7%), had received an influenza vaccine the previous year (231/251; 91.7%), and would recommend the COVID-19 vaccine to a family member (182/251; 72.2%).

The respondents had more differences in their attitudes regarding public health and health-protective measures (Table 2). For example, 163 nurses (64.7%) believed in the effectiveness of social distancing, and 143 (56.7%) thought that masks prevented the spread of COVID. Annual COVID-19 vaccination was supported by 137 nurses (54.4%), but some respondents (61/251; 24.2%) were uncertain about this possibility. A minority of respondents supported mandatory vaccines for healthcare workers (75/251; 29.8%). Reasons for not vaccinating were varied; the most common reason was concerned about side effects (n = 34; 85%), disagreement with mandates (n = 33; 82.5%), and previous infection with COVID-19 (n = 27; 67.5%) (Table 2). Text responses to some questions are recorded in Appendix B.

The characteristics of nurses who had received vaccination were analyzed by simple multivariable logistic regression analyses (Table 3). Nurses who thought masks were not effective (adjusted odds ratio {aOR]: 0.17; 95% confidence interval [CI] 0.04, 0.80), nurses who had not received an influenza vaccination (aOR: 0.04; 95% CI 0.00, 0.11), and nurses who would not recommend a COVID vaccine to a friend (aOR: 0.06; 95% CI 0.01, 0.22) were less likely to be vaccinated. Specifically, nurses who did not receive an influenza vaccine had a 99% decrease in the odds of having received a COVID-19 vaccine compared with those who received an influenza vaccine. Additionally, nurses who thought masks were not effective had an 83% decrease in the odds of having received a COVID-19 vaccine compared with those who thought masks were effective. The decrease was 86% for those who were unsure if masks were effective. In addition, compared to those who would recommend the COVID-19 vaccine to a friend, there was a 94% decrease in the odds of having received the COVID-19 vaccine among those who would not recommend the COVID-19 vaccine to a friend. The decrease was 92% among those who answered they were unsure. The odds of having received the COVID-19 vaccine seem to be lower for those in the 51–60 and >60 age groups. However, the differences were not statistically significant.

## 4. Discussion

This survey included 251 nurses who worked in a regional hospital that had a significant number of admissions with severe COVID-19 infections. Over this 24-month period, approximately 1200 patients were admitted to the medical ICU, which represents an average of 50 admissions per month. The majority of nurses exhibited pro-vaccine behavior; most received the COVID-19 vaccine, had prior vaccinations as children, received an annual influenza vaccine, and would recommend the COVID-19 vaccine to a friend or family member. A majority also supported public health measures such as social distancing and wearing masks during the pandemic and acknowledged the importance of receiving vaccines in general. However, only a minority supported vaccine mandates, with a majority opposed to such actions.

Multiple factors potentially influence the decisions of healthcare workers to undergo vaccination either during routine healthcare or during exceptional circumstances such as a pandemic. These factors include the healthcare workers’ assessment of their risk given any underlying medical conditions, their risk of infection at the worksite, the risk for transmission of any acquired infection to family, friends, or patients, their understanding of the efficacy of the vaccine in question, their understanding of the safety and potential side effects associated with the particular vaccine in question, and other potential social influences, such as religious beliefs and peer influences at the particular worksite [13]. Here, the most frequent reasons expressed involved concerns regarding the safety and effectiveness of the vaccine, the need for vaccination, and mistrust of/disagreement with government policy. In this survey, 84 nurses (40% of 211) who had received vaccination had not received a booster vaccine; 30 nurses (36% of 84) stated they did not plan to receive a booster vaccine, and 29 nurses (34.5%) stated they were unsure as to whether they would receive a booster vaccine. Follow-up surveys of this subgroup would provide additional information regarding vaccine hesitancy.

Interestingly, while a majority of nurses indicated pro-vaccine and pro-public health attitudes, most were opposed to vaccine mandates. Reasons for this may include libertarian political ideologies, emphasis on self-autonomy, mistrust of government, or perceived coercion. Some patients stated that they declined vaccination for religious or health reasons, with the misconception that the vaccines are produced from “fetal tissue” being cited by some recipients. COVID-19 vaccine hesitancy has also been correlated with political beliefs [14]. Some may refuse vaccination out of protest to these measures rather than an opposition to personally receiving the vaccine. Indeed, the second most frequent reason for not vaccinating was disagreement with vaccine mandates (n = 33, 82.5%). Concerns about safety and effectiveness are also frequently observed and are consistent with the published literature, which typically rates this as the area of greatest concern [4].

Healthcare worker vaccination status is of interest to hospitals and healthcare systems for patient and employee safety and meeting regulations regarding employee safety requirements. Several interventions have been studied for vaccine-hesitant HCWs, including email reminders, vaccine education, and monetary incentives and non-monetary incentives [15,16,17]. The hope is that these methods can address safety concerns, dispel misinformation, and provide incentives that can increase vaccination rates. Indeed, some healthcare workers have outlined specific circumstances, usually additional time to assess the vaccine’s safety, under which they would vaccinate [18,19]. However, addressing those who claim they will not vaccinate under any circumstances presents a unique challenge. As observed here, this includes a significant subset of those who chose not to vaccinate. The most frequent motivator to vaccinate (among the unvaccinated) was additional information about vaccine efficacy and safety. However, it is unclear to what extent this would be acceptable, given the amount of data already confirming that these vaccines are safe and effective. Future studies might consider exploring this desire for additional information in more depth. Finally, it should be noted that unvaccinated individuals most frequently receive information on COVID-19 vaccines from medical professionals and public health resources, although this study did not determine exactly what constitutes the former, and this may include the minority of healthcare professionals who are vocally opposed to the vaccine. It is also encouraging though perhaps surprising, that less reliable information sources such as YouTube, social media, blogs, and friends/family did not serve as information resources used by unvaccinated participants. Though this suggests that the HCWs are likely using reliable or reputable sources for vaccine information, it does not explain their decisions to ignore the pro-vaccine recommendations typically present in these sources. This may also provide an additional challenge to the perspective that merely providing reliable resources or eliminating disreputable ones will solve vaccine hesitancy.

Prior surveys of medical students, residents, faculty, and staff at this Health Sciences Center provide additional information about regional attitudes towards vaccination against COVID-19 infection. A survey was distributed to all Health Science Center employees in December 2020 before vaccines were available at this site [20]. In total, 2338 employees (51.8%) out of 4512 employees responded to this survey; 46% of the respondents indicated they definitely would receive a vaccine, 18% indicated they probably would receive a vaccine, 18% were uncertain, 7% indicated that they probably would not receive a vaccine, and 10% indicated that they would definitely not receive a vaccine. In this survey, 83% of the faculty indicated they probably would receive the vaccine. A second survey was distributed to residents and fellows at this institution in March 2021 [21]. Overall, 67 out of 81 respondents (82.7%) had received the vaccine. Of the 234 medical students who responded to a third survey distributed in July 2021, 215 students (91.9%) had been vaccinated [22]. Most students supported the use of COVID-19 booster vaccination and annual COVID-19 vaccination. Students who had not received a vaccine were waiting for more evidence of efficacy and had concerns regarding side effects. These three survey results demonstrate that medical students, postgraduate trainees, and faculty were likely to receive or had received COVID-19 vaccination.

Other studies have examined COVID-19 vaccination rates among HCWs. A scoping review of vaccine hesitancy among nurses across 51 studies and 41,098 nurses noted a hesitancy rate of 23.4% in 2020 which decreased to 18.3% for studies in 2021 [23]. A group of 32,426 active and retired nurses in the United States surveyed from March to June 2021 found that 93% had either received at least one dose of the vaccine or planned to do so. Furthermore, among hesitant respondents, concerns regarding safety (n = 1466; 67.0%), side effects (n = 1260; 57.6%), and efficacy (n = 699; 32.0%) were cited as the most frequent reasons for hesitancy [24]. This is consistent with what we observed in this study. A significant amount also stated a lack of concern regarding COVID-19 as a reason for hesitancy (n = 537; 24.6%) [24]. Finally, a study of vaccinated nurses found that 30.9% were hesitant to receive a second booster dose or a new COVID-19 vaccine formulation. As in other studies, the most common concerns among hesitant individuals involved safety (n = 45; 18.4%), side-effects (delineated between short- (n = 55; 22.4%) and long- (n = 115; 46.9%) term effects), and efficacy (n = 90; 36.7%) were the most common, which is intriguing given this group had already received COVID-19 immunization. This study also notes a unique reason for hesitancy: being fatigued with the “vaccination process” (n = 60; 24.5%) [25]. Similarly, this study observed a relatively low rate of booster vaccination (50% of vaccinated respondents) despite a much higher rate of overall vaccination, an observation that may warrant future studies.

Studies on vaccine uptake and vaccine hesitancy during a pandemic depend on the study timeframe in relation to the pandemic, and this study is no exception. The COVID-19 pandemic in the United States emerged in March 2020. At that point, the rates of infection, the rates of hospitalizations, and mortality rates were unknown. Over time it became apparent that this pandemic had extraordinary medical and social consequences. The first vaccine became available in the United States in December 2020. Experts concluded that herd immunity would require over an 80% participation rate by all people living in the United States. This created sustained efforts by government officials and experts in public health and infectious disease to educate the public to promote vaccination. In addition, vaccine mandates were contemplated by both private and public organizations. In September 2021, the Biden administration announced a COVID-19 action plan which would include mandates for a large portion of the American workforce. For example, all employees of all federally funded Medicaid and Medicare-certified healthcare facilities would require vaccination. This created significant public and private conversations regarding the legality of any mandate. In January 2022, the White House withdrew the mandate. Consequently, surveys regarding vaccine uptake and hesitancy clearly depend on the timeframe for the survey and its relationship to the ongoing pandemic, the availability of the vaccine, and the intense and widespread public discussion regarding the utility of vaccines and the legal basis for White House vaccine mandates. Indeed, one protest took place adjacent to the medical center where this study took place in objection to the vaccine mandate [26]. A large hospital in Houston, Texas (Houston Methodist) gained national attention for firing 215 employees who refused to comply with their institutional vaccine regulations [27]. Indeed, as observed here, some individuals will continue to resist vaccination. Hospitals will continue to need policies to clearly manage situations, need to maintain the best possible infection control measures, and need to respect the decisions made by informed healthcare workers.

This study has several limitations. The survey involved nurses in one regional hospital in West Texas who worked in a variety of settings in the hospital with various exposures to COVID-19 and critically ill patients. The survey response rate was relatively low, and the nurses who responded to the survey may have attitudes and opinions which do not necessarily reflect the “average” or prevailing attitude of these hospital employees. However, the rate of vaccine uptake by these nurses was similar to the rates reported by medical students and postgraduate trainees in this Health Sciences Center. In addition, surveys that ask about the intention for future vaccination may not provide good predictions regarding final vaccine uptake [28,29]. In some cases, survey respondents misrepresent their likelihood of future activity, and some survey respondents with good intention never take the vaccine. While this survey attempted to provide a comprehensive and non-judgmental list of potential reasons for not vaccinating, some individuals may not have fully read the list due to its length or felt that certain choices were not articulated sufficiently to represent their motivations. Free text responses were included with the anticipation that such individuals would provide answers not explicitly stated or appropriately articulated in the survey. Finally, this survey did not capture certain variables included in other studies, such as household living arrangements or income [5], which may be relevant to questions about vaccine hesitancy.

## 5. Conclusions

This survey indicates that most nurses working in this hospital received the COVID-19 vaccination and generally had pro-vaccine attitudes and behaviors. COVID-19 vaccination was associated with influenza vaccination, belief in the effectiveness of mask-wearing, and willingness to recommend the vaccine to a friend. Therefore, nurses may be a good resource for discussing vaccination with hospital employees, patients, and the public. A small percentage of nurses did not take the COVID-19 vaccine, with reasons for non-vaccination being varied. Organizations interested in promoting vaccination should consider the varied reasons for vaccine hesitancy when designing interventions. It also suggests that there is a small subset of HCWs who are highly resistant to vaccination. This presents a unique challenge to healthcare organizations that want to promote vaccination among their employees. Finally, reasons for HCW vaccine hesitancy are often complex and may produce or be driven by strong emotions. Those developing interventions or promoting vaccination should avoid promoting caricatures of those who are vaccine-hesitant and first recognize the reasons behind an individual’s hesitancy.

## Figures and Tables

**Table 1:** Basic demographics and answers to vaccination survey questions.

Variable	Category	N	%
Gender	Female Male Non-binary/non-conforming Prefer not to disclose	222 24 2 3	88.1 9.5 0.8 1.2
Age	20–30 31–40 41–50 51–60 >60	55 68 59 48 21	21.8 27.0 23.4 19.0 8.3
How many years have you practiced nursing?	Less than 1 1–5 6–10 11–15 16–20 21–25 >25	14 47 49 26 30 25 60	5.6 18.7 19.4 10.3 11.9 9.9 23.8
What is the highest level of nursing degree that you obtained?	Associate/certificate Bachelor’s Master’s Doctorate Other	71 118 45 3 14	28.2 46.8 17.9 1.2 5.6
Did you receive the COVID-19 vaccine?	Yes No	211 40	83.7 15.9
If you received the COVID-19 vaccine, which one did you receive?	Johnson & Johnson Moderna Pfizer Missing	2 80 128 1	1.0 37.9 60.7 0.5
If you received the Moderna or Pfizer COVID-19 vaccine, did you receive the second dose?	Yes No Missing	202 5 1	97.1 2.4 0.5
If you received the COVID-19 vaccine, have you received a booster dose?	Yes No Missing	126 84 1	59.7 39.8 0.5
If you have not received the COVID-19 booster dose, are you planning to receive one?	Yes No Unsure Missing	23 30 29 2	27.4 35.7 34.5 2.4
Did you receive any childhood vaccinations?	Yes, all of them Yes, some of them None Missing	242 6 2 1	96.0 2.4 0.8 0.4
Did you receive an influenza vaccine (“flu shot”) last year?	Yes No	231 20	92.0 7.9
Where do you receive information about COVID-19 vaccines? *	Public health website (e.g., CDC/local health department) Medical professionals Medical/academic journals News media Nursing professors/faculty Social media Friends/family Podcasts/radio talk shows YouTube Blogs Other	177 174 95 81 57 41 27 14 8 4 4	83.9 82.5 45.0 38.4 27.0 19.4 12.8 6.6 3.8 1.9 1.9
Where do you receive information about COVID-19 vaccines? **	Medical professionals Public health website (e.g., CDC/local health department) Medical/academic journals Podcasts/radio talk shows News media Nursing professors/faculty Social media Friends/family Other YouTube Blogs	34 27 26 16 14 9 5 5 5 2 1	85.0 67.5 65.0 40.0 35.0 22.5 12.5 12.5 12.5 5.0 2.5

* Received COVID-19 vaccine, ** Did not receive COVID-19 vaccine.

**Table 2:** Vaccine beliefs.

Variable	Category	N	%
Do you believe in the need to be vaccinated against other diseases (e.g., hepatitis B, measles, mumps, etc.)?	Yes No Unsure Missing	236 6 8 1	93.7 2.4 3.2 0.4
If an annual COVID-19 vaccine became available (similar to the annual influenza vaccine), would you plan to get it?	Yes No Unsure	137 53 61	54.4 21.0 24.2
Do you believe that vaccines should be mandated for healthcare workers?	Yes No Unsure	75 146 30	29.8 57.9 11.9
Do you believe that social distancing is effective at preventing the spread of COVID-19?	Yes No Unsure Missing	163 65 22 1	64.7 25.8 8.7 0.4
Do you believe that masks are effective at preventing the spread of COVID-19?	Yes No Unsure Missing	143 79 28 1	56.7 31.3 11.1 0.4
Would you recommend getting the COVID-19 vaccine to a friend or family member?	Yes No Unsure	182 40 29	72.2 15.9 11.5
If you chose not to receive the COVID-19 vaccine, please indicate why (select all that apply): *	- Concerned about side-effects - Disagree with vaccine mandates - Already had COVID-19 infection - Believe that natural infection/immunity is better - Vaccines were not studied enough - Do not believe the vaccine is effective - Disagree with vaccine portrayal in news media - Waiting until more evidence about the vaccine is available - Religious exemption - Believe that government or public health leaders are benefiting financially from the vaccine -Do not trust public health information about COVID-19/vaccinations - Believe that heard immunity is preferable to mass vaccination - Believe that diet/alternative medicine is better prevention for COVID-19 - Had a friend/relative who had a serious reaction to the vaccine - Vaccination no longer necessary at the current point in pandemic - Do not feel at risk for COVID-19 infection - Had a friend/relative who had a serious reaction to a vaccine (not COVID-19) - Had side effects from previous vaccine(s) (not COVID-19) - Other - Do not believe COVID-19 is a health risk - Not required to be vaccinated - Believe that masks/social distancing/hygiene are better prevention - Concerned vaccination would interrupt daily schedule	34 33 27 25 25 23 22 21 20 19 18 14 12 11 7 6 6 5 5 3 3 2 2	85.0 82.5 67.5 62.5 62.5 57.5 55.0 52.5 50.0 47.5 45.0 35.0 30.0 27.5 17.5 15.0 15.0 12.5 12.5 7.5 7.5 5.0 5.0
If you chose not to receive the COVID-19 vaccine, under which of the following circumstances would you consider receiving the vaccine? (select all that apply) *	- I do not plan on receiving the COVID-19 vaccine under any circumstances under any circumstances - Additional clinical data on vaccine safety/efficacy - Other - Required vaccination for employment - Financial incentive (e.g., bonus or reduced insurance premiums for vaccination) - New COVID-19 variants - Increase in COVID-19 cases/hospitalizations	24 15 5 3 3 2 1	60.0 37.5 12.5 7.5 7.5 5.0 2.5

* Percentage of those who did not receive COVID-19 vaccine (n = 40).

**Table 3:** Vaccination rates and nurse characteristics.

	Received (N = 211)	Not Received (N = 40)	Crude OR	Adjusted OR
	Number (%)	Number (%)	(95% CI)	(95% CI)
**Gender**				
Female	188 (89.10)	34 (85.00)	reference	reference
Male	20 (9.48)	4 (10.00)	0.90 (0.29, 2.81)	0.63 (0.10, 3.97)
Other	3 (1.42)	2 (5.00)	0.27 (0.04, 1.68)	0.03 (0.00, 1.16)
**Age**				
20–30	44 (20.85)	11 (27.50)	reference	reference
31–40	53 (25.12)	15 (37.50)	0.88 (0.37, 2.12)	1.63 (0.32, 8.25)
41–50	52 (24.64)	7 (17.50)	1.86 (0.66, 5.20)	24.82 (1.34, 460.1)
51–60	44 (20.85)	4 (10.00)	2.75 (0.81, 9.30)	0.83 (0.04, 19.51)
>60	18 (8.53)	3 (7.50)	1.50 (0.37, 6.02)	0.49 (0.01, 21.51)
**Years practiced**				
0–5	55 (26.07)	6(15.00)	reference	reference
6–10	35 (16.59)	14 (35.00)	0.27 (0.10, 0.78)	0.31 (0.06, 1.58)
11–15	20 (9.48)	6 (15.00)	0.36 (0.11, 1.26)	0.48 (0.04, 5.55)
16–20	23 (10.90)	7 (17.50)	0.36 (0.11, 1.18)	0.05 (0.00, 1.01)
21–25	22 (10.43)	3 (7.50)	0.80 (0.18, 3.48)	0.05 (0.00, 1.45)
>25	56 (26.54)	4 (10.00)	1.53 (0.41, 5.71)	1.85 (0.07, 49.16)
**Highest level of nursing degree**				
Associate/certificate	56 (26.54)	15 (37.50)	reference	reference
Bachelor’s	99 (46.92)	19 (47.50)	1.40 (0.66, 2.96)	1.51 (0.38, 5.97)
Master’s and above	42 (19.91)	6 (15.00)	1.88 (0.67, 5.24)	4.29 (0.45, 40.91)
Other	14 (6.64)	0 (0.00)	no estimable	no estimable
**Nursing title**				
Licensed Practical Nurse	29 (13.74)	4 (10.00)	reference	reference
Registered Nurse	173 (81.99)	32 (80.00)	0.75 (0.25, 2.27)	0.41 (0.07, 2.40)
Other	9 (4.27)	4 (10.00)	0.31 (0.06, 1.50)	0.08 (0.00, 1.51)
**Did you receive an influenza vaccine?**				
Yes	206 (97.63)	25 (62.50)	reference	reference
No	5 (2.37)	15 (37.50)	**0.04 (0.01, 0.12)**	**0.01 (0.00, 0.11)**
**Social distancing is effective?**				
Yes	150 (71.43)	13 (32.50)	reference	reference
No	42 (20.00)	23 (57.50)	**0.16 (0.07, 0.34)**	0.29 (0.07, 1.22)
Unsure	18 (8.57)	4 (10.00)	**0.39 (0.11, 1.32)**	0.72 (0.12, 4.39)
**Masks are effective?**				
Yes	138 (65.71)	5 (12.50)	reference	reference
No	50 (23.81)	29 (72.50)	**0.06 (0.02, 0.17)**	**0.17 (0.04, 0.80)**
Unsure	22 (10.48)	6 (15.00)	**0.13 (0.04, 0.47)**	**0.14 (0.02, 0.91)**
**Recommend the COVID-19 vaccine to a friend?**				
Yes	178 (84.36)	4 (10.00)	reference	reference
No	17 (8.06)	23 (57.50)	**0.02 (0.01, 0.05)**	**0.06 (0.01, 0.22)**
Unsure	16 (7.58)	13 (32.50)	**0.03 (0.01, 0.09)**	**0.08 (0.02, 0.30)**

Numbers in **bold** represent statistically significant differences.

## Data Availability

Data requests should be submitted to the corresponding author.

## Appendix A. Survey Questionnaire

1. Age
  - <20
  - 20–30
  - 31–40
  - 41–50
  - 51–60
  - >60
2. Gender
  - Female
  - Male
  - Transgender Female
  - Transgender Male
  - Non-binary/non-conforming
  - Not listed
  - Prefer not to disclose
3. How many years have you practiced nursing?
  - Less than 1
  - 1–5
  - 6–10
  - 11–15
  - 16–20
  - 21–25
  - >25
4. What is the highest level of nursing degree that you obtained?
  - Associate/certificate
  - Bachelor’s
  - Master’s
  - Doctorate
  - Other
5. What is your nursing title?
  - Licensed Practical Nurse (LPN)
  - Registered Nurse (RN)
  - Advanced Practice Registered Nurse (APRN)
  - Other [free text]
6. Did you receive the COVID-19 vaccine?
  - Yes
  - No
7. If you received the COVID-19 vaccine, which one did you receive?
  - Johnson & Johnson
  - Moderna
  - Pfizer
  - Other
8. If you received the Moderna or Pfizer COVID-19 vaccine, did you receive the second dose?
  - Yes
  - No
9. If you received the COVID-19 vaccine, have you received a booster dose?
  - Yes
  - No
10. If you have not received the COVID-19 booster dose, are you planning to receive one?
  - Yes
  - No
  - Unsure
11. If you chose not to receive the COVID-19 vaccine, please indicate why (select all that apply):
  - Concerned about side-effects
  - Don’t believe vaccine is effective
  - Don’t believe COVID-19 is a health risk
  - Don’t trust public health information about COVID-19/vaccinations
  - Vaccines weren’t studied enough
  - Had a friend/relative who had a serious reaction to the vaccine
  - Believe that natural infection/immunity is better
  - Do not feel at risk for COVID-19 infection
  - Believe that diet/alternative medicine is better prevention for COVID-19
  - Believe that herd immunity is preferable to mass vaccination
  - Believe that masks/social distancing/hygiene are better prevention
  - Believe that government or public health leaders are benefiting financially from the vaccine
  - Disagree with vaccine mandates
  - Disagree with vaccine portrayal in news media
  - Had side effects from previous vaccine(s) (not COVID-19)
  - Had a friend/relative who had a serious reaction to a vaccine (not COVID-19)
  - Concerned vaccination would interrupt daily schedule
  - Vaccine not available/too expensive
  - Waiting until more evidence about the vaccine is available
  - Already had COVID-19 infection
  - Not required to be vaccinated
  - Vaccination no longer necessary at current point in pandemic
  - Religious exemption
  - Other [free text]
12. If you have not received the COVID-19 vaccine, are you considering getting the COVID-19 vaccine?
  - Yes
  - No
  - Unsure
13. If you chose not to receive the COVID-19 vaccine, under which of the following circumstances would you consider receiving the vaccine?
  - New COVID-19 variants
  - Increase in COVID-19 cases/hospitalizations
  - Additional clinical data on vaccine safety/efficacy
  - Required vaccination for employment
  - No mask requirements for vaccinated individuals
  - Require vaccination for access to restaurants, bars, gyms, etc.
  - Financial incentive (e.g., bonus or reduced insurance premiums for vaccinated)
  - Other [free text]
14. Do you believe in the need to be vaccinated against other diseases (e.g., hepatitis B, measles, mumps, etc.)?
  - Yes
  - No
  - Unsure
15. Did you receive any childhood vaccinations?
  - Yes, all of them
  - Yes, some of them
  - None
  - Unsure
16. Did you receive an influenza vaccine (“flu shot”) last year?
  - Yes
  - No
  - Unsure
17. If an annual COVID-19 vaccine became available (similar to the annual influenza vaccine), would you plan to get it?
  - Yes
  - No
  - Unsure
18. Where do you receive information about COVID-19 vaccines? (Select all that apply)
  - Public health website (e.g., CDC)
  - News media
  - Social media
  - Podcasts/radio talk shows
  - Medical professionals
  - Medical/academic journals
  - Nursing professors/faculty
  - YouTube
  - Blogs
  - Friends/family
  - Other [free text]
19. Do you believe that vaccines should be mandated for healthcare workers?
  - Yes
  - No
  - Unsure
20. Do you believe that social distancing is effective at preventing the spread of COVID-19?
  - Yes
  - No
  - Unsure
21. Do you believe that masks are effective at preventing the spread of COVID-19?
  - Yes
  - No
  - Unsure
22. Would you recommend getting the COVID-19 vaccine to a friend or family member?
  - Yes
  - No
  - Unsure

Some questions only available based on participant responses.

## Appendix B. Free Text Responses to Questions

**Question 11: If you chose not to receive the COVID-19 vaccine, please indicate why.**
  - There is no way to know the 5–10-year (long-term) effects of the new mRNA vaccine method when this vaccine has NOT even been given to people for 5–10 years. I think a mass mandate of something we know little about is wrong (it is for me to choose what risk I am willing to take). The risk/benefit question does not have the same answer for everyone—a blanket coverage rule is not a smart idea—I am more concerned about the serious side effects of the vaccine for myself than dying from covid. That it not the same answer for everyone. Some are at more risk of dying from covid than from serious side effects from the vaccine. Let people who choose to get it. If my risks of dying of covid were much higher, I would have considered it to a higher degree (risk vs. benefit). My [relative] chose to take it after a discussion with his nephrologist. His risk is much higher than mine…If this were a new surgical procedure and one I could postpone without long-term consequences, I would wait till it had been widely done for at least 5 years. But if it were my only hope at improved life or maintaining what I had, I would be more willing to take the risk. I also believe it is important how it was developed. Because of the COVID-19 vaccine, I have learned other medicines may have had similar origins from fetal tissue. It is difficult for me to allow myself to benefit from the use of aborted fetuses, whether it is from yesterday or 100 years ago. All life is precious and to be respected. I would like to see medical researchers improve their ethics in the sources of early study and research. I myself am around covid frequently. I have had covid once. My symptoms were incredibly mild to nonexistent. I realize that is not everyone’s case, but for me, it is riskier to get a vaccine than to be happy with the natural immunity God gave me. I also have religious objections to the current vaccines. I would reconsider my stance after 5–10 years if my health changed, if a differently developed vaccine or more traditional vaccine were available, and…. This one came out far too fast for us to know the long-term safety of a new method. There are good ways to prevent the spread of contagious illnesses. Don’t leave home sick unless for healthcare. Social distance is wise. Hand hygiene etc. Masks help very little, but I can agree with them in limited circumstances like high current cases and in a healthcare facility. We need fresh air and sunshine for our health. Not everyone lives and works in a high-risk population.
  - I’m Immunocompromised
  - I would have to hold certain medications for weeks to take them.
  - Allergy to additives and the list of ingredients are not available to see if there is anything in it that would cause an anaphylactic reaction for me.
**Question 13: If you chose not to receive the COVID-19 vaccine, under which of the following circumstances would you consider receiving the vaccine?**
  - Whether the rule is to wear a mask or is a personal choice. Is it unwise to visually differentiate between vaxed and unvaxed. This would create division where there is none. We are a team. Let’s keep it that way. Just don’t come to work sick. If needed, do symptom check sheets. But all of us have the potential to get and spread covid. Though I have read that natural immunity speads illness less than vaccination. I have not seen other articles with info on this and would like to see more information.
  - Comorbidities or frequent close interaction with someone with comorbidities.
  - Application for RN school.
  - When the list of ingredients is made available, I will make my decision.
  - Looking for an alternative to the mRNA vaccines. Waiting for the held Pfizer documents to be released in their entirety.
**Question 18: Where do you receive information about COVID-19 vaccines?**
  - Dr Robert Malone
  - Church, Christian news broadcasts
  - Work
  - Weekly vaccine webinars through the state of Texas
  - Self-research of the data
  - Manufacturers websites
  - Workplace
